# Sedative Effects of Latexes Obtained from Some *Lactuca* L. Species Growing in Turkey

**DOI:** 10.3390/molecules25071587

**Published:** 2020-03-30

**Authors:** Selen Ilgün, Esra Küpeli Akkol, Mert Ilhan, Derya Çiçek Polat, Ayse Baldemir Kılıç, Maksut Coşkun, Eduardo Sobarzo-Sánchez

**Affiliations:** 1 Department of Pharmaceutical Botany, Faculty of Pharmacy, Erciyes Unıversity, 38039 Kayseri, Turkey; ilgunselen@gmail.com; 2 Department of Pharmacognosy, Faculty of Pharmacy, Gazi University, 06330 Ankara, Turkey; 3 Department of Pharmacognosy, Faculty of Pharmacy, Van Yüzüncü Yıl University, 65080 Tuşba/Van, Turkey; mertilhan@yyu.edu.tr; 4 Department of Pharmaceutical Botany, Faculty of Pharmacy, Ankara University, 06560 Ankara, Turkey; polatd@ankara.edu.tr (D.C.P.); mcoskun@ankara.edu.tr (M.C.); 5 Department of Pharmaceutical Botany, Gülhane Faculty of Pharmacy, University of Health Science, 06018 Ankara, Turkey; aysebaldemir@gmail.com; 6 Instituto de Investigación e Innovación en Salud, Facultad de Ciencias de la Salud, Universidad Central de Chile, Santiago 8330507, Chile; 7 Department of Organic Chemistry, Faculty of Pharmacy, University of Santiago de Compostela, 15782 Santiago de Compostela, Spain

**Keywords:** Asteraceae, *Lactuca*, sedative effect, sesquiterpene lactone, HPLC

## Abstract

*Lactuca* L. species belong to the Asteraceae family and these plants are traditionally used for therapeutic purposes around the world. The dried milky latex of *L. serriola* is known as “lettuce oil” and is used as a sedative in Turkey. This study aimed to evaluate the sedative effects and analyze the chemical compositions of latexes obtained from some *Lactuca* species growing in Turkey. The sedative effects were evaluated through various behavioral tests on mice. For this purpose, latexes were obtained from *L. glareosa* Boiss., *L. viminea* (L.) J. Presl and C. P, *L. mulgedioides* (Vis and Panćić) Boiss. and Kotschy ex. Boiss., *L. saligna* L., and *L. serriola* L. The latex from *L. saligna* showed the highest sedative effects, whilst *L. serriola* and *L. viminea* latexes also displayed significant sedative effects compared to the control group at a dose of 100 mg/kg. However, the latexes from *L. glareosa* and *L. mulqedioides* did not exhibit any sedative effects on mice. Characteristic sesquiterpene lactones (lactucin, lactucopicrin, 11,13β-dihydrolactucin, and 11,13β-dihydrolactucopicrin) were determined qualitatively and quantitatively by high-performance liquid chromatography (HPLC). Lactucin was identified as the main component.

## 1. Introduction

Sleep is the state of rest that is necessary for all people to lead healthy lives. Many physical, environmental, psychological, and physiological factors can positively or negatively affect the quality and quantity of sleep. Insomnia is considered an important health problem because of its negative effects on people’s quality of life and it often has psychiatric or medical causes [1]. Chronic sleep disorders are often defined as difficulties in initiating and maintaining sleep; consequently, the quality and quantity of sleep are low. Insomnia is often seen and treated as a symptom rather than a disease. Antidepressants and sedative and hypnotic drugs are used for insomnia, especially benzodiazepines, such as diazepam, which are sedative-hypnotic drugs used for the modulation of γ-aminobutyric acid (GABA) receptors [2]. The side effects of such hypnotic drugs make people uneasy about their use. Therefore, people often use herbal complementary therapies to treat insomnia and traditionally, many medicinal plants have been used for their sedative-hypnotic effects [3,4].

*Lactuca* is a taxon that contains important species and this genus is widely distributed throughout the world and has been consumed by people since ancient times. *L. biennis* (Moench) Fernald is used as an analgesic, antidiarrheic, antiemetic, and antihemorrhagic, as well as for heart diseases and gynecological diseases; *L. canadensis* L. is used as an analgesic, sedative, and stimulant and treats eye and kidney disorders, orthopedic diseases, skin diseases, and warts. *L. intybacea* Jacq. ex Murray is applied as a decongestant, laxative, tonic, and depurative, and treats arthritis, hepatitis, and gout. *L. muralis* (L.) Fresen is used as a narcotic, sedative, and antispasmodic. *L. sativa* L. is taken as an aphrodisiac, sedative, cardioactive, diuretic, hypnotic, narcotic, and sedative, and it treats asthma, fever, and hyperglycemia. *L. indica* L. is used for its antibacterial and anti-inflammatory effects. *L. capensis* Thunb is used for treating sores, ulcers, leprosy, eczema, and the milky latex of *L. virosa* L., known as “lactucarium”, is taken as a sedative-hypnotic [5,6,7]. Bioactivity studies on *Lactuca* species have shown that these plants have analgesic, anti-inflammatory, antioxidant, anticarcinogenic, hepatoprotective, neuroprotective, antidiabetic, and anxiolytic effects [8,9,10,11,12,13]. A review of the literature shows that sesquiterpene lactones are characteristic components of *Lactuca* species. In particular, lactucine-type guaianolides are the most representative constituents [14].

Lactucin and its esters were first found in *L. virosa* latex and they were specifically responsible for the sedative-hypnotic effects of lactucarium. Several studies on lactucin and its derivatives have confirmed that these compounds have sedative effects. In addition, it has been noted that lactucin and lactucopicrin are active substances that promote sleep, because they bind effectively to the GABA_A_ receptor [15,16].

The aim of this study was to evaluate the sedative effects of milky latexes obtained from five *Lactuca* species grown in Turkey. The sedative effects of the latexes obtained from *L. serriola, L saligna, L viminea, L. mulgedioides,* and *L. glareosa* were investigated using traction, fireplace, holeboard, and thiopental-induced sleeping tests. In addition, the presence and amounts of the sesquiterpene lactones lactucin and lactucopicrin and their dihydro derivatives were determined by high-performance liquid chromatography (HPLC). This is the first report on the sedative effects of latexes obtained from these *Lactuca* species.

## 2. Results

In Europe, the dried milky latex of wild lettuce (*L. virosa, L. serriola*) has traditionally been used as a sedative. In Turkey, the dried milky latex of *L. serriola* is known as “lettuce oil” and is used as a sedative [17]. In this study, the sedative effects of latexes obtained from five *Lactuca* species grown in Turkey were investigated by various in vivo methods on mice. The latexes were also analyzed using high-performance liquid chromatography for characteristic compounds thought to be responsible for this effect.

The sedative-hypnotic effects of the latexes on mice were determined by traction, fireplace, holeboard, and thiopental-induced sleep tests. These behavioral tests showed that the latexes from *L. saligna*, *L. serriola,* and *L. viminea* had significant sedative effects compared to the control group at a dose of 100 mg/kg whilst the latexes from *L. glareosa* and *L. mulqedioides* did not exhibit any sedative effects (Table 1).

Another method that can be used to evaluate sedative effects is the thiopental-induced sleep test. The results of this test showed that the latexes from *L. saligna, L. serriola,* and *L. viminea* significantly reduced the time to the onset of sleeping and increased the sleeping time of mice compared to the control group (Table 2).

The latex obtained from *L. saligna* showed the highest activity followed by the latex from *L. serriola.*

**Figure 1:**
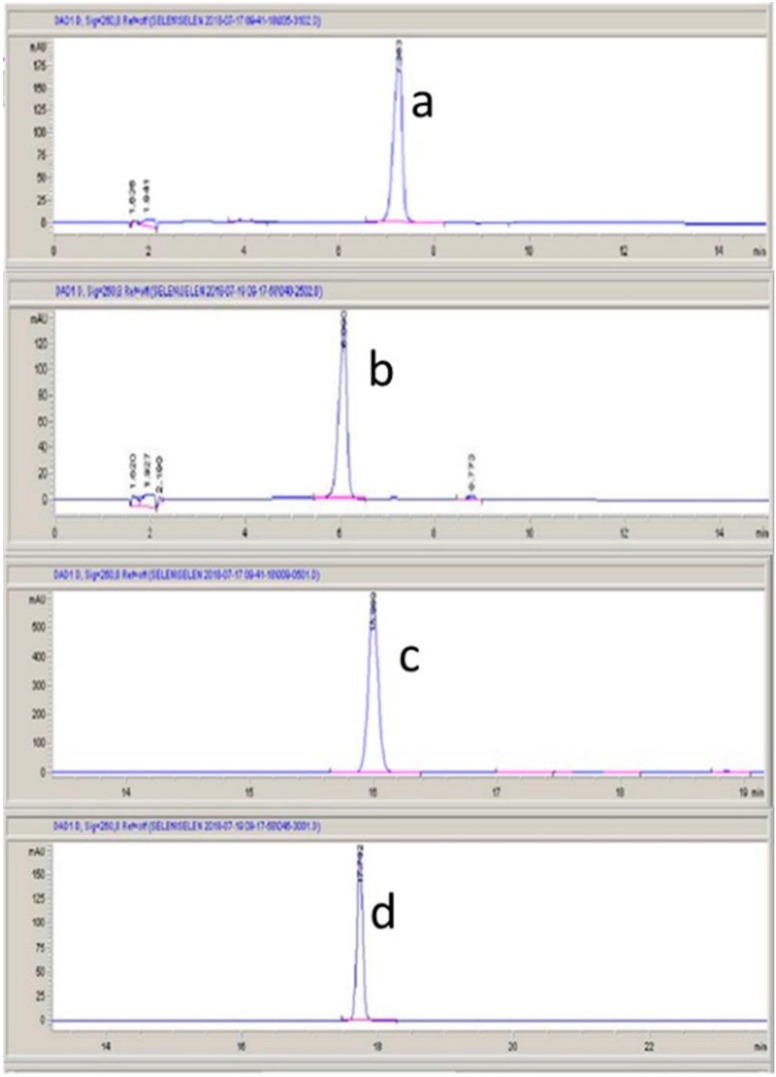
High-performance liquid chromatography (HPLC) chromatograms of the standards: (**a**) lactucin, (**b**) 11β,13-dihydrolactucin, (**c**) lactucopicrin, and (**d**) 11β,13-dihydrolactucopicrin.

The latexes were evaluated for lactucin-type guaianolides (lactucin, lactucopicrin, 11β,13-dihydrolactucin, and 11β,13-dihydrolactucopicrin), which are characteristic constituents of the *Lactuca* genus. According to the literature, these compounds have sedative effects, which have been shown in various studies and attempts have been made to prove this effect in both in vivo and in vitro experiments [4,15,16]. The constituents were separated using HPLC and quantified by a diode-array detector (DAD). Figure 1 shows the HPLC chromatograms of the standards.

**Figure 2:**
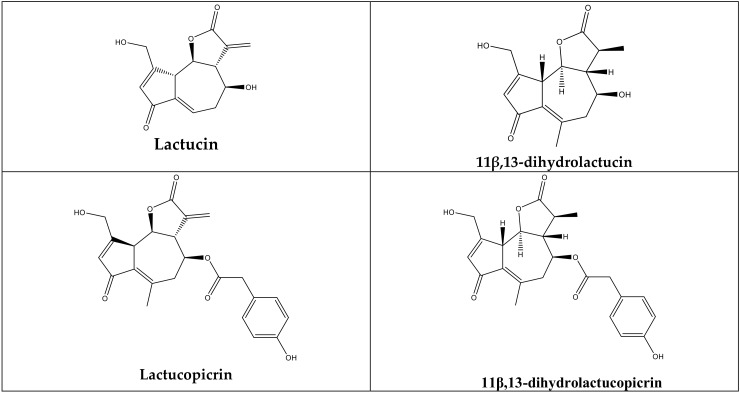
Molecular formulas of the sesquiterpene lactones.

**Figure 3:**
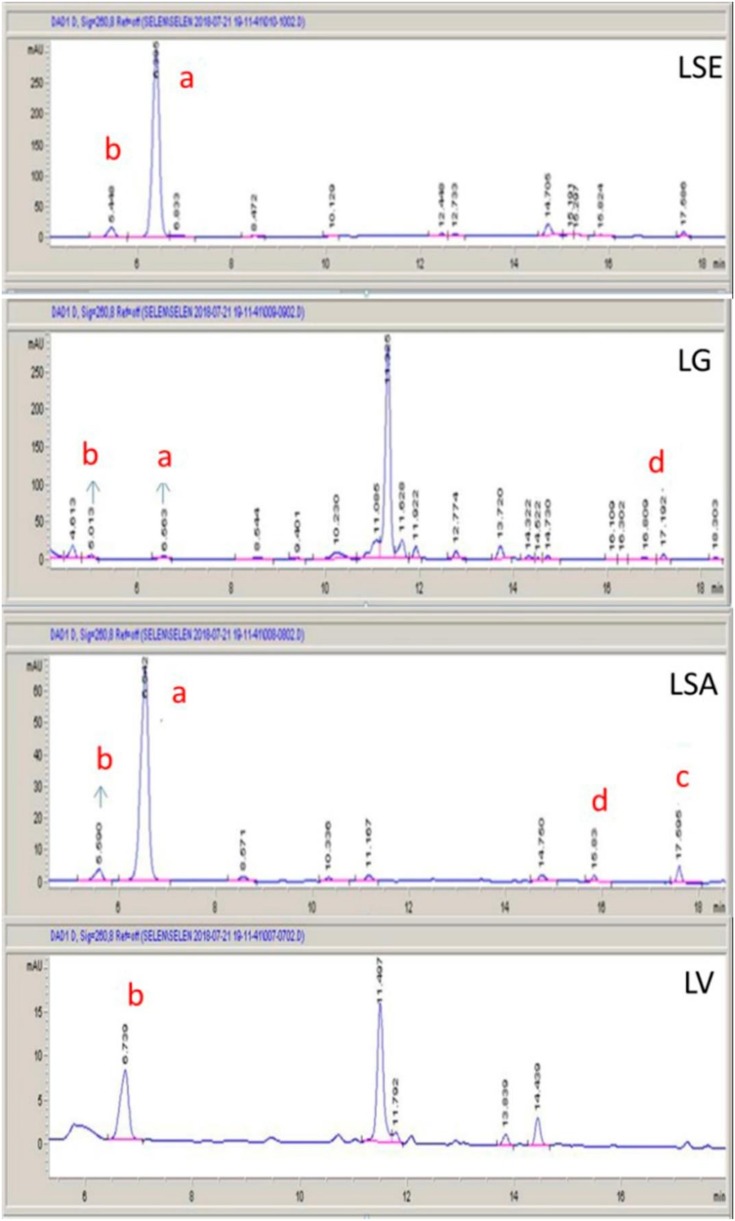
HPLC chromatograms of the latexes. LSE: *L. serriola* latex, LG: *L. glareosa* latex, LSA: *L. saligna* latex, LV: *L. viminea* latex. Peaks: (**a**) lactucin, (**b**) 11β,13-dihydrolactucin, (**c**) lactucopicrin, and (**d**) 11β,13-dihydrolactucopicrin.

Sesquiterpene lactones were identified by retention times. Quantification was achieved by the measurement of the peak area at 260 nm and by comparison with standard calibration curves (Table 3). The highest lactucin content was found in the latex from *L. serriola* (57.53249 ± 0.27 mg_std_/g_latex_)*,* followed by *L. saligna, L. viminea*, and *L. glareosa* in descending order. Lactucopicrin was only detected in the latex from *L. saligna* (0.61729 ± 0.02 mg std/g_latex_) and all the other reference compounds were also found in the latex from *L. saligna* (Figure 2 and Figure 3). The 11β,13-dihydro derivatives of lactucin and lactucopicrin were found in the latexes from *L. glareosa, L. saligna*, and *L. serriola*. The highest level of 11β,13-dihydrolactucin was found in the latex from *L. serriola* (2.35356 ± 0.03 mg _std_/g_latex_) whilst the highest level of 11β,13-dihydrolactucopicrin was seen in the latex from *L. glareosa* (0.75238 ± 0.01 mg _std_/g_latex_). In *L. mulgedioides*, none of the compounds of interest was detected.

Although the amount of lactucin detected in *L. serriola* was considerably higher than the amount of lactucin detected in *L. saligna*, the sedative effect of the latex from *L. serriola* was lower than that of the latex from *L. saligna*.

## 3. Discussion

*Lactuca* species are used in folk medicine for stomach problems, pain relief, and inflammation. Furthermore, it has been reported to have anticonvulsant, sedative-hypnotic, and antioxidant properties [18]. Sutrisna also reported significant analgesic, anti-inflammatory, antidepressant, and anti-coagulant properties and found that the 70% ethanolic extract of the leaves of *L. sativa* displayed a remarkable sedative effect [19]. Additionally, Kim et al. showed that the 70% ethanolic extract of *L. sativa* enhanced sleeping time and acted via the GABAergic system [16].

In the literature, the quantitative analysis of sesquiterpene lactones has been reported for various species of *Lactuca* genus. However, few studies have been reported with latexes from *Lactuca* species. The latexes of *L. mulgedioides, L. saligna, L. viminea*, and *L. glareosa* were examined for the first time in this study. Sessa et al. noted that important information about the sesquiterpene lactone content of the latex of *L. sativa*, was obtained and they reported that the major sesquiterpene lactones of *Lactuca* species are 15-oxalyl and 8-sulfate conjugates of guaianolides, such as lactucin, deoxylactucin, and lactucopicrin [20]. In this present work, lactucin derivatives were identified in the methanolic extracts of the latexes obtained from the studied plants by their characteristic UV spectra.

The literature shows that different amounts of latex are found in different vegetation stages of the plant. It has been reported that the sesquiterpene lactone content is highest during bolting. However, in our study, all the latexes were collected during the flowering stage because the amount of latexes of plants was high at this stage. The lactucin amounts were compared with the literature and a significant difference was observed between *L. sativa* and our plant material, *L. serriola* [20]. The results of this showed that the age and growth stage of the plant are important parameters affecting the amount of sesquiterpenes.

The sedative effects of 70% ethanolic extracts of the leaves and seeds of different varieties of *L. sativa* were investigated using the pentobarbital-induced sleeping test by Kim et al. and the amounts of lactucin and lactucopicrin in the extracts were determined by HPLC. A stronger sedative effect was observed in the seed extract of *L. sativa* which contained more lactucopicrin [4]. In another study, whilst lactucin and lactucopicrin showed sedative effects, 11β,13-dihydrolactucin did not show any effect at doses of 15 mg/kg and 30 mg/kg. Moreover, lactucin and lactucopicrin have been reported as the main active sedative substances from spontaneous locomotor activity tests [15]. In addition, the sleep-enhancing effects of lettuce were investigated, and the extracts of lettuce inhibited the binding of [3H]-flumazenil in a concentration-dependent manner and the affinity of both lactucin and lactucopicrin to gamma-aminobutyric acid (GABA)A-benzodiazepine (BDZ) receptor was 80.7% and 55.9%, respectively. Thus, lettuce extracts have been reported to act through a GABAergic mechanism to promote sleep [16].

According to our study results, in order to understand the mechanism of the effects of the compounds, further studies should be carried out. Our results showed that the sedative effect is not caused by lactucin, because the latex of *L. serriola* (LSE) whose lactucin content was significantly higher (57.53249 ± 0.27, mg _std_/g_latex_) had no higher sedative effect. The lactucopicrin compound was found only in *L. saligna* (LSA) and a high sedative effect was observed in this latex. In *L. viminea* (LM), no components we analyzed were seen in this latex and accordingly no sedative effect was obtained. These results confirm that lactucin-type guaianolides are responsible for the sedative effects.

We can understand from these results that lactucin and lactucopicrin likely have a synergistic effect in increasing sedative activity. Moreover, other components of latexes that we did not detect may also be responsible for the sedative effect. Therefore, in-depth structural determination and characterization studies should be carried out for all of the main components of latex, which will help elucidate the compound or compounds responsible for the sedative effect.

## 4. Materials and Methods

### 4.1. Plant Materials and Collection of Latex

Firstly, various *Lactuca* species were collected from different habitats in Central Anatolia during the flowering period from July to September of 2018 for the extraction of latex. The obtained plants (*L. serriola*, *L. saligna, L. glareosa*, and *L. mulgedioides*) were authenticated by Prof. Dr. Ergin Hamzaoglu from Gazi University.

The stems of the plants were wounded with a scalpel and the milky latex was released onto the plant surface. The latex was then collected in glass bottles. This process was repeated three times and the samples were stored at –20 °C until the moment of the analysis.

### 4.2. HPLC Analysis

The collected latex samples were extracted with methanol and centrifuged at 16,000× *g* for 10 min. The obtained supernatant was then filtered through a 0.45 µm membrane filter. The extracted latexes were analyzed by liquid chromatography using an Agilent 110 (G1379A, Santa Clara, CA, USA).

Samples were injected into a reverse-phase (RP) C_18_ column (15 cm × 4.6 mm, 5 μm) with a CH_3_CN/H_2_O gradient over 40 min at 1 mL min^−1^ and 40 °C. The solvent mixture consisted of acetonitrile (solvent A) and water (solvent B). The portion of A was increased from 12% to 95% in 37 min gradually, then returned to the initial conditions in 2 min and the percentage of B was decreased from 88% to 5% in 37 min and then returned to the initial conditions.

### 4.3. Bioactivity Tests

#### 4.3.1. Animals

Male BALB/c mice (25–30 g) were obtained from Kobay Animals Laboratory (Ankara, Turkey). The animals were allowed to adapt to the laboratory conditions for 3 days before the experiment. In this process, the animals were housed with a 12-h light, 12-h dark cycle with standard laboratory chow and tap water.

Each test group consisted of six mice and all the studies were performed conferring to the international rules regarding the animal experiments and biodiversity rights (Kobay Ethical Council Project Number: 314).

#### 4.3.2. Administration of Test Materials

In all the tests, samples were administered intraperitoneally in a 0.5% sodium carboxymethylcellulose (CMC) suspension at a dose of 100 mg/kg. In the control group, only 0.5% CMC was administered.

Lorazepam (Sigma, CAS No: 846-49-1, Darmstadt, Germany) was used as a reference drug and it was administered to the mice intraperitoneally at a dose of 1 mg/kg.

#### 4.3.3. In Vivo Tests in Mice

##### Traction Test

The traction test was applied to the mice to evaluate the sedative effects of the test materials using the method described by Courvoisier with Laroche and Rousselet [21,22,23,24].

The test samples and lorazepam as a reference material were administered to the mice intraperitoneally. An hour later, the mice were placed horizontally with their forepaws on a taut rope. Normally a mouse hanging on the rope will lift its hind legs, whilst mice that fail to lift at least one of their hind legs to reach the rope are considered to be sedated. In addition, the behavior of the animals was recorded during the experimental period.

##### Fireplace Test

The method developed by Hoffman was applied to the mice [25,26].

The test samples and lorazepam as a reference material were administered to the mice intraperitoneally. An hour later, each mouse was placed in a 30-cm long vertical glass tube. Typically, a normal mouse attempts to escape within thirty seconds, whilst mice that do not make any attempt during this period are considered to be sedated.

##### Holeboard Test

The method developed by Clark et al. and File and Hoffman was applied to the mice [27,28].

The test samples and lorazepam as a reference material were administered intraperitoneally to the mice. An hour later, the mice were placed in the center of a perforated board. The perforated board test was carried out using a wooden board of 40 × 40 × 25 cm with evenly spaced holes. The number of times the mice inserted their heads into the holes was recorded.

##### Thiopental-Induced Sleeping Test

The method developed by Aziz and Khan was applied to the mice [29,30,31].

Thiopental (a sub-hypnotic dose) was administered to the mice (60 mg/kg) intraperitoneally, then after 30 min the mice were treated with the test samples. The time from the application of the test samples to sleep and the time from sleep to waking was recorded.

##### Statistical Analysis of the Data

The data obtained from the animal experiments were evaluated using one-way ANOVA statistical tests. In addition, the test and control groups were assessed using Student–Newman–Keuls post hoc tests. The results were compared with those from the control and reference groups. Statistical significance is expressed as * *p* < 0.05, ** *p* < 0.01, and *** *p* < 0.001.

## Figures and Tables

**Table 1:** Sedative effects of test materials determined by the traction test, fireplace test, and holeboard test.

Test Material	Dose (mg/kg)	Traction Test (s) (Re-Establishment Time) Mean ± SEM	Fireplace Test (s) (Time to Go Back the Tube in Seconds) Mean ± SEM	Holeboard Test (Explored Holes during 5 min) Mean ± SEM
Control	-	0.11 ± 0.00	9.14 ± 0.87	34.11 ± 1.86
LG	100	2.15 ± 0.19	12.56 ± 1.22	33.47 ± 1.18
LM	100	1.05 ± 0.06	10.28 ± 0.93	31.06 ± 1.93
LSA	100	7.83 ± 0.29 **	131.84 ± 1.41 ***	6.21 ± 0.52 **
LSE	100	5.34 ± 0.37 *	93.99 ± 0.98 **	15.64 ± 0.97 *
LV	100	4.21 ± 0.14 *	75.99 ± 1.04 *	27.58 ± 1.10
Lorazepam	1	10.27 ± 0.43 ***	168.39 ± 1.35 ***	0.00 ± 0.00 ***

* *p* < 0.05; ** *p* < 0.01; *** *p* < 0.001; LSE: *L. serriola* latex, LG: *L. glareosa* latex, LSA: *L. saligna* latex, LV: *L. viminea* latex, LM: *L. mulgedioides* latex. -: 0.5% CMC

**Table 2:** Sedative effects of test materials determined by the thiopental-induced sleeping test.

Test Material	Dose (mg/kg)	Onset of Sleeping (min) Mean ± SEM	Sleeping Duration (min) Mean ± SEM
Control	-	54.11 ± 1.98	60.28 ± 2.47
LG	100	49.53 ± 2.75	78.61 ± 2.80
LM	100	52.81 ± 2.90	66.49 ± 2.64
LSA	100	20.13 ± 1.71 ***	224.26 ± 2.52 ***
LSE	100	24.77 ± 1.93 *	205.91 ± 2.87 **
LV	100	36.15 ± 2.01 *	173.66 ± 2.98 *
Lorazepam	1	17.43 ± 1.54 ***	297.15 ± 2.83 ***

* *p* < 0.05; ** *p* < 0.01; *** *p* < 0.001; LSE: *L. serriola* latex, LG: *L. glareosa* latex, LSA: *L. saligna* latex, LV: *L. viminea* latex, LM: *L. mulgedioides* latex. -: 0.5% CMC

**Table 3:** HPLC analysis of the sesquiterpene lactones in the latexes from *Lactuca* species.

Materials	Lactucin (mg _std_/g_latex_)	Lactucopicrin (mg _std_/g_latex_)	11β,13 Dihydrolactucopicrin (mg _std_/g_latex_)	11β,13 Dihydrolactucin (mg _std_/g_latex_)
LG	0.45551 ± 0.01 *	-	0.75238 ± 0.01 *	0.55975 ± 0.1 *
LM	-	-	-	-
LSA	13.94970 ± 0.24 *	0.61729 ± 0.02 *	0.49266 ± 0.01 *	0.47723 ± 0.01 *
LSE	57.53249 ± 0.27 *	-	-	2.35356 ± 0.03 *
LV	1.40352 ± 0.01 *	-	-	-

LSE: *L. serriola* latex, LG: *L. glareosa* latex, LSA: *L. saligna* latex, LV: *L. viminea* latex, LM: *L. mulgedioides* latex. * mean ± SD; SD: standard deviation; *n* = 3. (-: No peak of the related compound was seen in the HPLC chromatogram)
